# Supplementary material for: Endogenous Polyamines and Ethylene Biosynthesis in Relation to Germination of Osmoprimed Brassica napus Seeds under Salt Stress
Source: Int J Mol Sci. 2021 Dec 29;23(1):349. doi: 10.3390/ijms23010349 (PMC8745725; doi:10.3390/ijms23010349)
Supplement: Supplementary file 1 [file ijms-23-00349-s001.zip › ijms-1517146-supplementary.pdf]

*Supplementary Material*

# Endogenous Polyamines and Ethylene Biosynthesis in Relation to Germination of Osmoprimed *Brassica napus* Seeds under Salt Stress

**Katarzyna Lechowska, Łukasz Wojtyla, Muriel Quinet, Szymon Kubala, Stanley Lutts and Małgorzata Garnczarska ***

**Table S1.** PAs concentrations (nmol/g DW) in the seeds of *Brassica napus* before the start of germination (dry unprimed seeds. $UP_d$ and primed dried seeds. $P_d$) as well as during germination (primed seeds germinating 7 h in water. $P_{7H2O}$; unprimed seeds germinating 11 h in water. $UP_{11H2O}$; primed seeds germinating 7 h in NaCl. $P_{7NaCl}$; unprimed seeds germinating 16 h in NaCl. $UP_{16NaCl}$).

| PAs | $UP_d$ | $P_d$ | $P_{7H2O}$ | $UP_{11H2O}$ | $P_{7NaCl}$ | $UP_{16NaCl}$ |
|---|---|---|---|---|---|---|
| Free Put | 45.73 ± 0.68 | 53.20 ± 4.77 | 61.23 ± 5.86 | 43.10 ± 3.96 | 60.80 ± 2.39 | 62.93 ± 2.46 |
| Conjugated Put | 57.63 ± 5.17 | 68.90 ± 2.57 | 54.33 ± 5.06 | 43.35 ± 1.44 | 53.74 ± 1.36 | 85.16 ± 2.53 |
| Bound Put | 36.06 ± 2.09 | 38.95 ± 0.90 | 38.60 ± 3.55 | 38.77 ± 4.21 | 26.33 ± 1.96 | 29.32 ± 1.36 |
| **Total Put** | **139.42 ± 2.68** | **161.05 ± 6.08** | **154.16 ± 7.85** | **125.22 ± 2.31** | **140.87 ± 2.98** | **177.41 ± 1.91** |
| Free Spd | 515.58 ± 4.90 | 396.00 ± 30.60 | 431.03 ± 40.01 | 425.79 ± 18.00 | 430.64 ± 3.39 | 328.04 ± 1.80 |
| Conjugated Spd | 519.15 ± 29.80 | 502.25 ± 41.13 | 350.15 ±15.71 | 420.56 ± 13.88 | 339.61 ± 25.50 | 471.85 ± 4.18 |
| Bound Spd | 382.39 ± 23.24 | 315.93 ±30.96 | 344.59 ± 26.58 | 370.81 ± 49.75 | 259.08 ± 22.84 | 265.07 ± 9.67 |
| **Total Spd** | **1417.12 ± 11.24** | **1214.18 ± 57.47** | **1125.77 ± 39.21** | **1217.16 ± 66.25** | **1029.33 ± 18.26** | **1064.96 ± 7.41** |
| Free Spm | 141.38 ± 4.10 | 112.74 ± 9.75 | 122.72 ± 15.45 | 111.36 ± 4.99 | 126.50 ± 6.21 | 82.48 ± 1.82 |
| Conjugated Spm | 116.23 ± 17.98 | 127.22 ± 5.77 | 88.41 ± 6.11 | 92.47 ± 5.32 | 99.73 ± 10.36 | 109.33 ± 2.29 |
| Bound Spm | 35.31 ± 3.21 | 29.81 ± 3.02 | 37.06 ± 2.09 | 41.19 ± 3.15 | 26.65 ± 3.01 | 23.77 ± 2.48 |
| **Total Spm** | **292.92 ± 18. 37** | **269.77 ± 19. 58** | **248.19 ± 21.71** | **245.02 ± 12.29** | **252.88 ± 6.95** | **215.58 ± 4.54** |
| **Free PAs** | **702.69 ± 7.41** | **561.94 ± 34.94** | **614.98 ± 41.27** | **580.25 ± 36.62** | **617.94 ± 10.13** | **473.45 ± 2.10** |
| **Conjugated PAs** | **693.01 ± 52.37** | **698.37 ± 50.10** | **492.90 ± 21.64** | **556.38 ± 23.29** | **493.08 ± 33.23** | **666.34 ± 5.64** |
| **Bound PAs** | **453.76 ± 24.29** | **384.69 ± 11.77** | **420.25 ± 14.15** | **450.77 ± 32.45** | **312.06 ± 20.36** | **318.16 ± 0.30** |
| **Total PAs** | **1849.46 ± 32.11** | **1645.00 ± 82.51** | **1528.12 ± 65.53** | **1587.40 ± 80.63** | **1423.08 ± 21.91** | **1457.95 ± 5.35** |
